# Supplementary material for: CD4 count and tuberculosis risk in HIV-positive adults not on ART: a systematic review and meta-analysis
Source: PeerJ. 2017 Dec 14;5:e4165. doi: 10.7717/peerj.4165 (PMC5733368; doi:10.7717/peerj.4165)
Supplement: Supplemental Information 2 [file peerj-05-4165-s002.docx]

# CD4 count and tuberculosis risk in people living with HIV not on ART: a systematic review and meta-analysis.

P.K. ELLIS^1,*^, W.J. MARTIN^1,*^, and P.J. DODD, Ph.D^2,#^

1. University of Sheffield Medical School, Sheffield, UK
2. School of Health and Related Research, University of Sheffield,
   Regent Court, 30 Regent Street, Sheffield, S1 4DA, UK

^*^ joint first authors, contributed equally to this work

^#^ corresponding author, email: p.j.dodd@sheffield.ac.uk T: +441142220740 F: +441142220749

**The rationale for conducting the meta-analysis**

Infection with the human immunodeficiency virus (HIV) is the strongest known risk factor for incidence of active tuberculosis (TB). Risk of TB incidence in people living with HIV (PLHIV) who are not receiving antiretroviral therapy (ART) is known to depend strongly on decline in CD4 cell count. CD4 cell count is a widely used proxy for increases in TB risk in mathematical models used for planning, resource allocation, and burden estimation. Typically this has been modelled as an exponential relationship between CD4 cell decrement and the risk of incident TB. The parameter defining this relationship is commonly based on a weighted average of a non-systematic review of cohorts of PLHIV. Given the importance of this parameter, we undertook a systematic review of cohorts of PLHIV not on ART in whom TB incidence had been reported stratified by CD4 cell count. We performed a statistically rigorous meta-analysis of our systematic review data in order to generate an updated estimate of this frequently used modelling parameter.

**The contribution that the meta-analysis makes to knowledge in light of previously published related reports, including other meta-analyses and systematic reviews**

This is the first meta-analysis accounting for between-study variation that has been applied to this question. It is the only meta-analysis of data obtained via systematic review, and restricted to cohorts with repeat measures of CD4 cell count. In these respects it is a methodological improvement over the previous weighted average estimate of this quantity from a non-overlapping set of studies. Our estimated value confirms the point estimate used in the modelling literature and produces more rigorous (and wider) uncertainty bounds.

For references, please see the main paper.
