# Supplementary material for: CD4 count and tuberculosis risk in HIV-positive adults not on ART: a systematic review and meta-analysis
Source: PeerJ. 2017 Dec 14;5:e4165. doi: 10.7717/peerj.4165 (PMC5733368; doi:10.7717/peerj.4165)
Supplement: Appendix S1 [file peerj-05-4165-s003.docx]

# Appendix - CD4 count and tuberculosis risk in people living with HIV not on ART: a systematic review and meta-analysis.

P.K. ELLIS^1,*^, W.J. MARTIN^1,*^, and P.J. DODD^2,#^

1. University of Sheffield Medical School, Sheffield, UK
2. School of Health and Related Research, University of Sheffield, UK

^*^ joint first authors, contributed equally to this work

^#^ corresponding author, email: p.j.dodd@sheffield.ac.uk

## Supplementary methods

### Full search strategy

Table 1: Full MEDLINE search strategy

| **Search** | **Search term** |
| --- | --- |
| 1 | exp Tuberculosis/ |
| 2 | ((tuberculosis or TB) adj5 (diagnos* or aetiology or etiology or association or connection or link or patholog* or risk or incidence or relation* or correlat*)).mp . [mp=title, abstract, original title, name of substance word, subject heading word, keyword heading word, protocol supplementary concept word, rare disease supplementary concept word, unique identifier] |
| 3 | exp HIV/ |
| 4 | (HIV or Human Immunodeficiency Virus or AIDS or Acquired Immune Deficiency Syndrome or HIV1 or HIV2).mp . [mp= title, abstract, original title, name of substance word, subject heading word, keyword heading word, protocol supplementary concept word, rare disease supplementary concept word, unique identifier] |
| 5 | CD4 Lymphocyte Count/ |
| 6 | (CD4 or CD-4 or T Lymphocyte).mp . [mp= title, abstract, original title, name of substance word, subject heading word, keyword heading word, protocol supplementary concept word, rare disease supplementary concept word, unique identifier] |
| 7 | 1 or 2 |
| 8 | 3 or 4 |
| 9 | 5 or 6 |
| 10 | 7 and 8 and 9 |
| 11 | limit 10 to (english language and humans and (“adolescent (13 to 18 years)” or “young adult (19 to 24 years)” or “adult (19 to 44 years)” or “young adult and adult (19 to 44 years)” or “middle age (45 to 64 years)” or “middle aged (44 plus years)” or “all aged (65 and over)” or “aged (80 and over)”)) |

### Modified Newcastle-Ottawa scale

Note: A study can be awarded a maximum of one star for each numbered item within the Selection and Outcome categories. A maximum of two stars can be given for Comparability

This analysis involved within-cohort comparisons and so control questions were not applicable.

Selection

S1) Representativeness of the people with HIV and and a given CD4 count

a) Truly representative of the average person with HIV and a given CD4 count

in the community **⍟**

b) Somewhat representative of the average person with HIV and a given CD4 count

in the community **⍟**

c) Selected group of people e.g. those admitted to hospital, being seen in clinic etc.

d) No description of the representativeness of the cohort

S2) Selection of non-exposed individuals *[cross-cohort comparisons and obtain no star]*

S3) Ascertainment of HIV status

a) Result taken from laboratory records **⍟**

b) Result obtained from medical records or structured patient interview **⍟**

c) self report

d) No description

S4) Demonstration that the individual did not have TB at the start of the study

a) Yes **⍟**

b) No

Comparability

C1) Comparability of cohorts on the basis of the design or analysis

a) The study controls for age of people when comparing different CD4 counts **⍟**

b) Study controls for other factors such as gender, socioeconomic status, BCG vaccination status, nutritional status, etc. **⍟**

Outcome

O1) Assessment of incident TB disease

a) Microbiologically confirmed TB disease **⍟**

b) Clinically diagnosed TB disease using defined criteria **⍟**

c) Clinician decision to treat for TB disease

d) No description made

O2) Was follow-up long enough for outcomes to occur

a) Yes, follow-up was for over a year after recruitment in the majority of people **⍟**

b) No, follow-up often short or not described

O3) Adequacy of follow up of cohorts

a) Complete follow up - all subjects accounted for **⍟**

b) Subjects lost to follow up unlikely to introduce bias - small number lost to follow-up (<20%), or description provided of those lost **⍟**

c) Loss to follow-up >20% and no description of those lost

d) No statement

### Statistical model specification for meta-analysis

Let $I_{ij}$ be the $j$-th incidence measurement for the $i$-th study, and $c_{ij}$ the corresponding CD4 count (measured relative to a study-dependent offset $f_{i}$). We assume a hierarchical model:

$$log(I_{ij})\sim N(\alpha_{i}.c_{ij}+\beta_{i},s_{ij}^{2})$$

$$\alpha_{i}\sim N(\alpha,\tau^{2})$$

$$\beta_{i}\sim N(0,s_{\beta}^{2})$$

$$\alpha\sim halfN(0,5^{2})$$

$$\tau\sim halfN(0,5^{2})$$

$$s_{\beta}\sim halfN(0,{50}^{2})$$

where the precision of each measurement $s_{ij}^{2}$ are derived from confidence intervals, and $halfN$ is the half-normal distribution. For the studies reporting hazard ratios with respect to a fixed CD4 count, we make the offset $f_{i}$ the CD4 value used as a reference value for the hazard ratios, and drop the reference incidence (i.e. 1, with infinite precision). For studies reporting incidence, we take $f_{i}\equiv0$ (differences in background TB incidence are accounted for by the intercepts $\beta_{i}$).

### R & Stan code & data for meta-analysis model

library(rstan)

## data

cd4datx <-

structure(list(N = 19L, K = 7L, y = c(1.15057202759882, 1.05431202977153, 0.215111379616945, 0.496640988774034, 0.105889756210005, -0.891472470364648, -1.3655245996335, -2.49192740108114, 0.182321321499865, -0.693147180559945, 1.64865862558738, 0.431782416425538, 4.09774015122324, 3.68887945411394, 3.09698600725916, 1.86254864082079, 2.3859667019331, 1.41881755282545,

-8.51719319141624),

sigma = c(0.338487484859462, 0.378044967066087, 0.0555417050624669, 0.230606868106005, 0.160430031848201, 0.166731743242249, 0.202038911645575, 0.325654819581601, 0.257921405911729, 0.286055796648826, 0.240104198938037, 0.214336887376643, 0.134536655244755, 0.125036492698743,

0.0801432728851067, 0.0507657466351781, 0.503138307205871, 0.503138307205871,

1.54684809394403),

cd4 = c(175, 425, 0, 25, 75, 275, 425, 750, 100, 600, 100, 275, 25, 75, 150, 600, 100, 350, 750),

offset = c(750, 50, 0, 0, 600, 0, 0),

hsn = 3L, hn = 5L,

HRdp = c(1L, 2L, 3L, 11L, 12L),

Jdp = c(4L, 5L, 6L, 7L, 8L, 9L, 10L, 13L, 14L, 15L, 16L, 17L, 18L, 19L),

H = c(1L, 1L, 2L, 5L, 5L),

J = c(3L, 3L, 3L, 3L, 3L, 4L, 4L, 6L, 6L, 6L, 6L, 7L, 7L, 7L),

Jb = c(1L, 1L, 1L, 1L, 1L, 2L, 2L, 3L, 3L, 3L, 3L, 4L, 4L, 4L)),

.Names = c("N", "K", "y", "sigma", "cd4", "offset", "hsn", "hn", "HRdp", "Jdp", "H", "J", "Jb"))

## stan model definition

stnmod <- "

data {

int<lower=0> N; // number of observations

int<lower=0> K; //number of studies

int<lower=0> hsn;//no HR studies

int<lower=0> hn;//no HR data points

int<lower=0> HRdp[hn];//HR data points

int<lower=0> Jdp[N-hn];//incidence data points

int<lower=0> H[hn];//HR data points study

int<lower=0> J[N-hn];//incidence data points study

int<lower=0> Jb[N-hn];//incidence data points study, offset mapping

real y[N]; // estimated treatment effects

real cd4[N]; // associated cd4 count

real offset[K]; // study offsets (ref pts for HRs)

real<lower=0> sigma[N]; // s.e. of effect estimates

}

parameters {//

real Alph; //summary alpha

real<lower=0> tau; //sd of alpha

real<lower=0> betasig; //sd of background log-inc

real alpha[K]; //individual study gradients

real beta[K-hsn]; //study background log-inc (except HRs)

}

model {

//priors

betasig ~ normal(0,50);//half-normal with above constraint

tau ~ normal(0,5); //half-normal with above constraint

Alph ~ normal(0,5);

//study intercepts

for(i in 1:(K-hsn))

beta[i] ~ normal(0,betasig);

//study gradients

for(i in 1:K)

alpha[i] ~ normal(Alph,tau);

//---likelihood

//data for HR studies with offsets (needed as 1 point serves as reference w/0 CI)

for(i in 1:hn)

y[HRdp[i]] ~ normal( alpha[H[i]] * (cd4[HRdp[i]] - offset[H[i]]), sigma[HRdp[i]]);

//data for incidence studies

for(i in 1:(N-hn))

y[Jdp[i]] ~ normal( beta[Jb[i]] + alpha[J[i]] * (cd4[Jdp[i]] - offset[J[i]]), sigma[Jdp[i]]);

}

"

## running stan model

fitx <- stan(model_code = stnmod,data = cd4datx,iter = 5000, chains = 4,control = list(adapt_delta = 0.999))

## Supplementary results

### Modified Newcastle-Ottawa quality assessment

Table 2: Quality assessment answers to specific questions (no. stars & items awarded for [in brackets])

|  | **SELECTION** | | | | **COMPARABILITY** | **OUTCOME** | | | **TOTAL** |
| --- | --- | --- | --- | --- | --- | --- | --- | --- | --- |
| **Question** | 1 | 2 | 3 | 4 | 1 | 1 | 2 | 3 |  |
|  |  |  |  |  |  |  |  |  |  |
| **Max score** | ⍟ | ⍟ | ⍟ | ⍟ | ⍟⍟ | ⍟ | ⍟ | ⍟ | 9 |
|  |  |  |  |  |  |  |  |  |  |
| Assebe, 2015 | 1 (B) | NA | 0 | 1 (A) | 2 (A+B) | 1 (B) | 1 (A) | 0 | 6 |
|  |  |  |  |  |  |  |  |  |  |
| Collins, 2015 | 1 (B) | NA | 0 | 1 (A) | 2 (A+B) | 1 (A) | 1 (A) | 1 (B) | 7 |
|  |  |  |  |  |  |  |  |  |  |
| Grant, 2009 | 1 (A) | NA | 1 (B) | 1 (A) | 1 (B) | 0 | 1 (A) | 1 (B) | 6 |
|  |  |  |  |  |  |  |  |  |  |
| Markowitz, 1997 | 1 (A) | NA | 0 | 0 | 1 (B) | 1 (B) | 1 (A) | 1 (B) | 5 |
|  |  |  |  |  |  |  |  |  |  |
| Monge, 2014 | 1 (B) | NA | 0 | 1 (A) | 2 (A+B) | 1 (A) | 0 | 0 | 5 |
|  |  |  |  |  |  |  |  |  |  |
| Nicholas, 2011 | 1 (B) | NA | 1 (B) | 1 (A) | 2 (A+B) | 1 (A) | 0 | 1 (B) | 7 |
|  |  |  |  |  |  |  |  |  |  |
| Wolday, 2003 | 0 | NA | 1 (A) | 0 | 2(A+B) | 1 (B) | 1 (A) | 0 | 5 |
|  |  |  |  |  |  |  |  |  |  |

### MCMC outputs


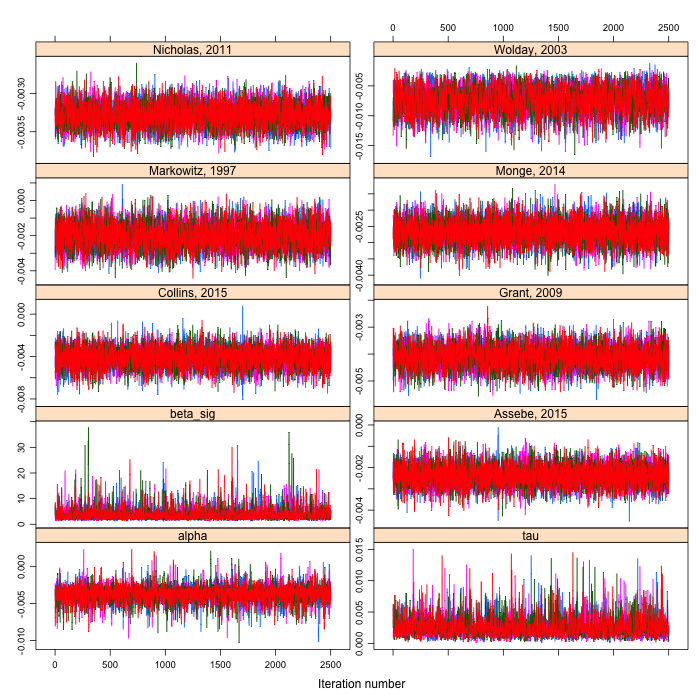


Figure 1: MCMC chains: Log-gradients for individual studies, summary log-gradient (alpha) and standard deviation (tau), standard deviation for study offsets (beta_sig); 4 chains for 2,500 iterations after warm-up 2,500

Table 3: Posterior parameter means (log-gradients are per CD4 cell/mm^3^)

| **Parameter** | **Mean** | **SD** | **Naive SE** | **Time-series SE** |
| --- | --- | --- | --- | --- |
| alpha | -0.003693 | 0.0011303 | 1.13E-05 | 1.51E-05 |
| tau | 0.002443 | 0.0014734 | 1.47E-05 | 2.73E-05 |
| beta_sig | 4.021394 | 2.7325363 | 2.73E-02 | 4.88E-02 |
| Assebe 2015 | -0.002372 | 0.000516 | 5.16E-06 | 5.05E-06 |
| Collins 2015 | -0.004084 | 0.001007 | 1.01E-05 | 9.76E-06 |
| Grant 2009 | -0.004104 | 0.0004383 | 4.38E-06 | 5.51E-06 |
| Markowitz 1997 | -0.002049 | 0.0007378 | 7.38E-06 | 9.45E-06 |
| Monge 2014 | -0.002665 | 0.0003781 | 3.78E-06 | 3.58E-06 |
| Nicholas 2011 | -0.003275 | 0.0001601 | 1.60E-06 | 1.90E-06 |
| Wolday 2003 | -0.007277 | 0.00238 | 2.38E-05 | 4.32E-05 |

Table 4: Posterior parameter means: sensitivity analysis with the zero incidence represented as lower 1/1,000^th^ of lower confidence interval rather than 1/100^th^ above (log-gradients are per CD4 cell/mm^3^)

| **Parameter** | **Mean** | **SD** | **Naive SE** | **Time-series SE** |
| --- | --- | --- | --- | --- |
| alpha | -0.004109 | 0.0017211 | 1.72E-05 | 2.58E-05 |
| tau | 0.003813 | 0.0020028 | 2.00E-05 | 3.61E-05 |
| beta_sig | 4.297453 | 3.0007014 | 3.00E-02 | 5.48E-02 |
| Assebe 2015 | -0.002304 | 0.0005158 | 5.16E-06 | 4.67E-06 |
| Collins 2015 | -0.00425 | 0.0010415 | 1.04E-05 | 8.73E-06 |
| Grant 2009 | -0.004138 | 0.000435 | 4.35E-06 | 4.91E-06 |
| Markowitz 1997 | -0.001888 | 0.0007603 | 7.60E-06 | 8.88E-06 |
| Monge 2014 | -0.002636 | 0.0003794 | 3.79E-06 | 3.53E-06 |
| Nicholas 2011 | -0.003278 | 0.0001613 | 1.61E-06 | 1.82E-06 |
| Wolday 2003 | -0.010234 | 0.0024566 | 2.46E-05 | 4.42E-05 |

### Funnel plot

Figure 2: Funnel plot to assess publication bias.
